# In Silico Study, Physicochemical, and In Vitro Lipase Inhibitory Activity of *α*,*β*-Amyrenone Inclusion Complexes with Cyclodextrins

**DOI:** 10.3390/ijms22189882

**Published:** 2021-09-13

**Authors:** Luana Carvalho de Oliveira, Danielle Lima Bezerra de Menezes, Valéria Costa da Silva, Estela Mariana Guimarães Lourenço, Paulo Henrique Santana Miranda, Márcia de Jesus Amazonas da Silva, Emerson Silva Lima, Valdir Florêncio da Veiga Júnior, Ricardo Neves Marreto, Attilio Converti, Euzébio Guimaraes Barbosa, Ádley Antonini Neves de Lima

**Affiliations:** 1 Pharmacy Department, Federal University of Rio Grande do Norte, Natal 59012-570, RN, Brazil; luanacoliver@ufrn.edu.br (L.C.d.O.); daniellelbmenezes@gmail.com (D.L.B.d.M.); vcs.biomed@gmail.com (V.C.d.S.); estela.mariana@hotmail.com (E.M.G.L.); paulxvii@hotmail.com (P.H.S.M.); euzebiogb@gmail.com (E.G.B.); 2 Biological Activity Laboratory, Pharmacy Department, Federal University of Amazonas, Manaus 69077-000, AM, Brazil; marciajas24@gmail.com (M.d.J.A.d.S.); eslima75@gmail.com (E.S.L.); 3 Chemistry Department, Military Engineering Institute, Rio de Janeiro 22290270, RJ, Brazil; valdir.veiga@gmail.com; 4 Pharmacy Department, Federal University of Goiás, Goiás 74605-170, GO, Brazil; ricardomarreto@ufg.br; 5 Department of Civil, Chemical and Environmental Engineering, University of Genoa, I-16145 Genoa, Italy; converti@unige.it

**Keywords:** amyrenone, triterpenes, cyclodextrins, inclusion complexes, lipase activity

## Abstract

*α*,*β*-amyrenone (ABAME) is a triterpene derivative with many biological activities; however, its potential pharmacological use is hindered by its low solubility in water. In this context, the present work aimed to develop inclusion complexes (ICs) of ABAME with *γ*- and *β*-cyclodextrins (CD), which were systematically characterized through molecular modeling studies as well as FTIR, XRD, DSC, TGA, and SEM analyses. In vitro analyses of lipase activity were performed to evaluate possible anti-obesity properties. Molecular modeling studies indicated that the CD:ABAME ICs prepared at a 2:1 molar ratio would be more stable to the complexation process than those prepared at a 1:1 molar ratio. The physicochemical characterization showed strong evidence that corroborates with the in silico results, and the formation of ICs with CD was capable of inducing changes in ABAME physicochemical properties. ICs was shown to be a stronger inhibitor of lipase activity than Orlistat and to potentiate the inhibitory effects of ABAME on porcine pancreatic enzymes. In conclusion, a new pharmaceutical preparation with potentially improved physicochemical characteristics and inhibitory activity toward lipases was developed in this study, which could prove to be a promising ingredient for future formulations.

## 1. Introduction

*α*,*β*-Amyrenones (ABAME) are triterpenoid isomers of the ursan and oleanan series that occur naturally in low concentrations in various oleoresins of *Protium* (Burseraceae) species widespread in the Brazilian Amazon or can be obtained by the oxidation of *α*,*β*-amyrin isolated from the oleoresin mainly of *Protium heptaphyllum* [1].

Although ABAME and compounds of the same class have a wide range of proven biological activities, they present in its crystalline state unfavorable physicochemical characteristics for pharmaceutical applications such as a poor solubility in water, which can reduce their bioavailability [1].

Oral administration of ABAME in mice resulted in a reduction in mechanical hypersensitivity and carrageenan-induced paw and ear edema; in addition, inhibition of nitric oxide and interleukin 6 (IL-6) production and increased synthesis of IL-10 in lipopolysaccharide-stimulated murine J774 macrophages were observed [1]. In addition, the triterpene extract of *Cnidoscolus chayamansa* containing *β*-amyrenone showed significant anti-inflammatory activity [2].

Hypoglycemic and hypolipemic actions of *P. heptaphyllum* oleoresin triterpenes have been previously reported [3,4]. ABAME has already been described as having potential in the treatment of chronic and metabolic diseases because it displayed inhibitory effects in vitro on lipase, *α*-glucosidase, and *α*-amylase [1,5]; therefore, it is necessary to evaluate its hypoglycemic activity and the mechanisms of action involved in this activity [6]. Thus, a methodology similar to that of current studies on antidiabetic and anti-obesity action was followed, focusing on the activity of the mixture against digestive enzymes involved in the metabolism of lipids and glycides. These studies demonstrated that the *P. heptaphyllum* resin, which contains the triterpene portion of the *α*,*β*-amyrenone mixture, was able to reduce the levels of oral and postprandial glycemia, in addition to stimulating body weight loss in rats by the mechanism of inhibition of digestive enzymes [1,4,7,8,9,10].

Obesity is a modern problem that grows exponentially, so much so that health authorities are unable to keep track of the growth in the number of obese people around the world. A good alternative may be drugs that act against pancreatic lipase, which is the key enzyme for the absorption of lipids that catalyzes the hydrolysis of triacylglycerols in the gastrointestinal tract; in fact, it is generally thought that a potent and specific inhibitor of pancreatic lipase may be useful in the treatment of obesity [11].

In view of ABAME physical and chemical limitations, it is important to develop soluble intermediates and evaluate their biological activities in in vivo models. The preparation of cyclodextrin complexes is a technological strategy that is widely used to increase the solubility, dissolution, stability, and bioavailability of poorly water-soluble compounds [12]. Cyclodextrins are cyclic starch-derived oligosaccharides that contain six, seven, eight, or more units of D-glycopyranose joined by an *α*-1,4 bond. Compounds containing six, seven, and eight monomeric units, known as *α*-cyclodextrin (*α*CD), *β*-cyclodextrin (*β*CD), and *γ*-cyclodextrin (*γ*CD), respectively, have low toxicity due to their reduced absorption in the gastrointestinal tract and complete metabolization by colon microflora [9,13].

CDs have a supramolecular structure that looks similar to a cage, similar to crypts, calixarenes, cyclophanes, and spherands. Their conical shape has openings of larger and smaller diameter formed by secondary and primary hydroxyl groups, respectively [14]. Due to such a specific arrangement, the outer part is sufficiently hydrophilic to give aqueous solubility to CDs, while the interior is considerably hydrophobic, being accessible to hydrophobic guest molecules of appropriate size [15].

Based on this background, the objective of the present work was to develop inclusion complexes with cyclodextrins for ABAME delivery, aiming at obtaining a new system capable of improving solubility and biological activity compared to the isolated compound, as well as its potential therapeutic use.

## 2. Results

### 2.1. Theoretical Computational Studies

#### 2.1.1. Physicochemical Properties In Silico (SwissADME)

**Figure 1:**
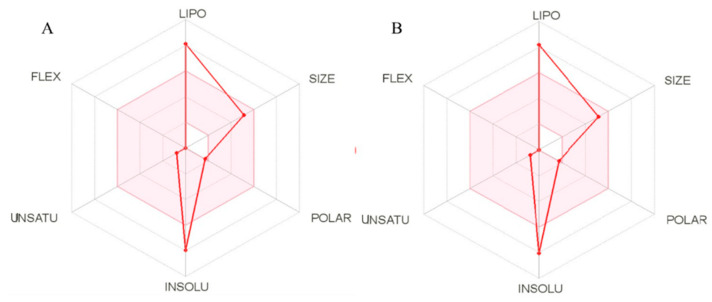
Solubility profiles of *α*- (**A**) and *β*- (**B**) amyrenones. Lipo (Lipid affinity), Size (Molecular structure size), Polar (Polarity of the molecule), Insolu (Compound water insolubility), Unsatu (Compound unsaturations), Flex (Structural flexibility).

To be effective as a drug, a molecule must reach its biological target in a satisfactory concentration and remain in the bioactive form long enough for the expected biological events to occur. However, data on the behavior of bioactive molecules such as *α*,*β*-amyrenone are limited. In this context, we used a SwissADME web tool [16] capable of providing information on the physicochemical and pharmacokinetic properties of the compounds. The binary mixture of *α*,*β*-amyrenone has low solubility in water (Figure 1A,B). It is possible to observe that the *α*- and *β*-isomers have a profile similar to that exhibited by poorly soluble oily resin.

ABAME isomers are made up of a carbon chain containing several rings. These structures are derived from the extraction of an oleoresin, which explains its high lipophilicity (log P) and low solubility in water (log S) (Table 1). Two topological methods used to predict water solubility are included in SwissADME: the ESOL 36 model and the model adapted by Ali et al. [17]. Both differ from the traditional general solubility equation, since they avoid using the melting point as a parameter in order to improve the test virtual reliability. A third solubility predictor was developed by SILICOS-IT to obtain a consensus between the means. The three models were calculated by the OpenBabel relating the values of log S and log P (Table 1). The models were translated into a qualitative estimate of the solubility class, according to the following scale of log S: insoluble <−10, <slightly soluble <−6, <moderately soluble <−4, <soluble <−2, <very soluble <0, <highly soluble.

As can be seen in Table 1, the final value of mean lipophilicity (log P) was 7.14, which means that the compound is hydrophobic, corroborating the literature and its origin derived from an oleoresin. As expected, the compound was characterized by low water solubility (log S), which justifies the efforts made in this study to improve this property in order to achieve satisfactory systemic availability.

Druglikeness assesses whether a compound can behave as a good drug using five parameters that suggest its viability or not, with the value 0 indicating a compound close to the ideal drug and 5 indicating an unfeasible product with the need for major modifications. The compound investigated in this study (ABAME) showed only one to two violations in each parameter, which indicates that it is promising for a future formulation.

#### 2.1.2. Virtual Screening

**Figure 2:**
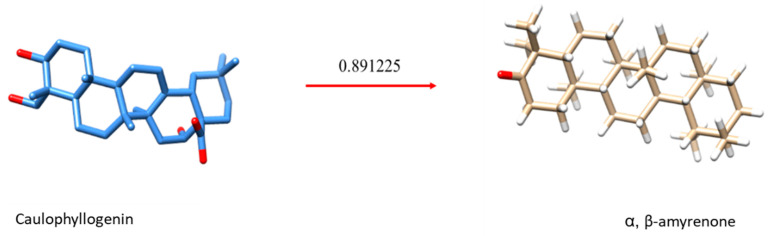
Similarity between the caulophyllogenin and *α*,*β*-amyrenone triterpenes.

In an attempt to elucidate a possible mechanism of action of the binary mixture, virtual simulations were carried out in a database with molecules with already clarified mechanisms. Similarity was observed between *α*,*β*-amyrenone and caulophyllogenin (Figure 2), which is a triterpene extracted from the species *Caulophyllum robustum* [18]. These compounds showed a similarity score with the PPAR-*γ* receptor (Table 2), which is also the target receptor for the drug ORLISTAT^®^ and acts either as an agonist, inhibiting the action of pancreatic lipases, or as a blocker of about 30% of the fats ingested by the patient (“[Pharmacological treatment of obesity]—PubMed,” n.d.). This virtual screening indicates that *α*,*β*-amyrenone could be used in the anti-obesity treatment.

#### 2.1.3. Molecular Docking

**Figure 3:**
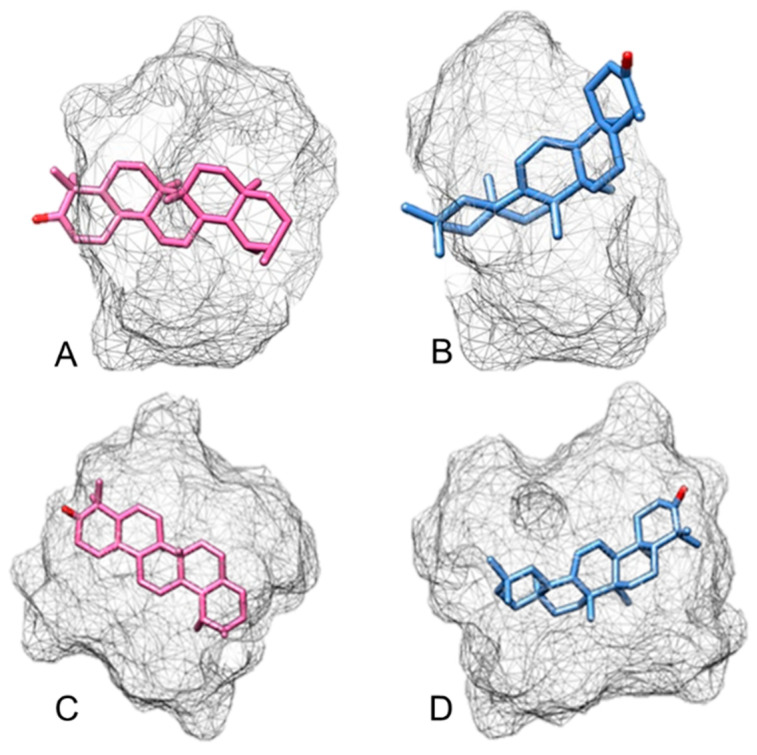
Energetic simulation of the interaction between *α*,*β*-amyrenone and *β*-cyclodextrin (*β*CD) in the molar ratios of 1:1 (**A**,**B**) and 2:1 (**C**,**D**). (**A**) *α*-amyrenone: *β*CD, coupling with the face containing hydroxyl groups inside the *β*CD cavity, (**B**) *β*-amyrenone: *β*CD, coupling with the face containing the hydroxyl groups outside the *β*CD cavity, (**C**) *α*-amyrenone: *β*CD in complete coupling, (**D**) *β*-amyrenone: *β*CD, in complete coupling.

**Figure 4:**
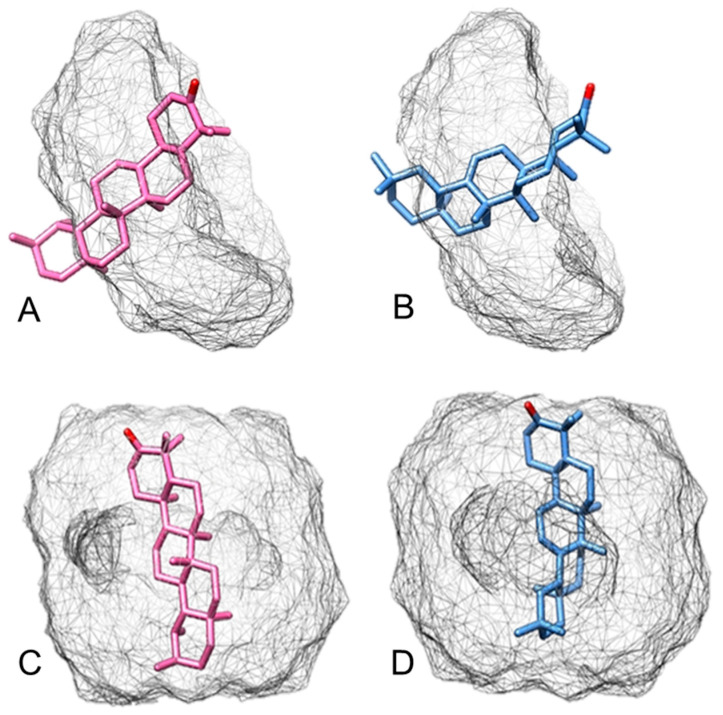
Energetic simulation of the interaction between *α*,*β*-amyrenone and *β*-cyclodextrin (*γ*CD) in the molar ratios of 1:1 (**A**,**B**) and 2:1 (**C**,**D**). (**A**) *α*-amyrenone: *γ*CD, coupling with the face containing hydroxyl groups inside the *γ*CD cavity, (**B**) *β*-amyrenone: *γ*CD, coupling with the face containing the hydroxyl groups outside the *γ*CD cavity, (**C**) *α*-amyrenone: *γ*CD in complete coupling, (**D**) *β*-amyrenone: *γ*CD, in complete coupling.

The theoretical computational study was carried out by separately simulating *α*-amyrenone and *β*-amyrenone complexes with *β*CD and *γ*CD, in the 1:1 and 2:1 (CD:active ingredient) molar ratios, in order to in silico evaluate intermolecular interactions between *β*CD:ABAME (Figure 3) and *γ*CD:ABAME (Figure 4). This type of evaluation makes it possible to reduce costs and predict the most appropriate molar ratio to enhance complexation and, consequently, to maximize ABAME water solubility.

In Figure 3A,B, the interaction between the components is shown in the 1:1 (*β*CD:active ingredient) molar ratio. In particular, Figure 3A shows the inclusion of *α*-amyrenone in the *β*CD cavity with the face containing hydroxyl groups, implying a minimum interaction energy of −39.55 kcal mol^−1^. Similarly, Figure 3B shows *β*-amyrenone coupling with the face containing hydroxyls outside the cavity, resulting in a minimum energy of −40.93 kcal mol^−1^. On the other hand, Figure 3C,D show the complete coupling of *α*-amyrenone and *β*-amyrenone in the 2:1 molar ratio, with minimum energies of −112.46 and −106.88 kcal mol^−1^, respectively. Even if there is a good molecular interaction in the 1:1 molar ratio, both *α*- and *β*-amyrenone are completely filled by CDs in the 2:1 ratio; therefore, taking into account the energy values listed in Table 3, it can be suggested that the latter ratio would give greater stability to the complex with *β*CD.

Figure 4A,B show a simulation of the inclusion of *α*-amyrenone and *β*-amyrenone in *γ*CD at a 1:1 molar ratio, respectively. The interaction of both active ingredients with the *γ*CD cavity occurs through the face containing hydroxyl groups, with minimum interaction energies of −23.38 and −18.76 kcal mol^−1^, respectively. On the other hand, the simulations in the 2:1 ratio are shown in Figure 4C,D for *α*-amyrenone and *β*-amyrenone, respectively. It is possible to observe that the coupling of compounds can occur by the two faces, with interaction energies of −81.83 and −81.92 kcal mol^−1^ (Table 3), respectively, showing that, similar to what was observed with *β*CD, the 2:1 ratio would give greater stability also to complexes prepared with *γ*CD.

### 2.2. Physicochemical Characterization

#### 2.2.1. Powder X-ray Diffraction

**Figure 5:**
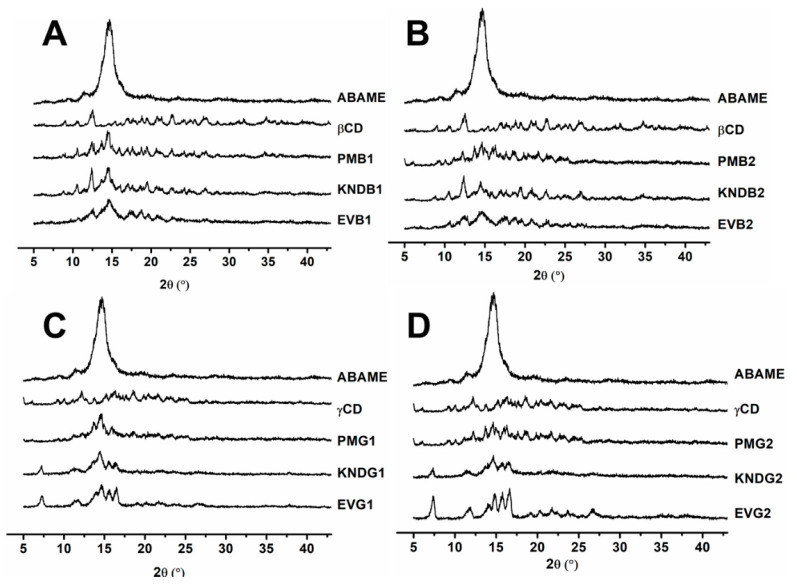
X-ray diffraction profiles of individual compounds, namely *α*,*β*-amyrenone (ABAME), *β*-cyclodextrin (*β*CD) and *γ*-cyclodextrin (*γ*CD), as well as inclusion complexes (ICs). ICs prepared with ABAME and *β*CD at 1:1 (**A**) and 2:1 (**B**) molar ratios using physical mixture (PMB1 and PMB2), kneading (KNDB1 and KNDB2), and rotary evaporation (EVB1 and EVB2), respectively. ICs prepared with ABAME and *γ*CD at 1:1 (**C**) and 2:1 (**D**) molar ratios using physical mixture (PMG1 and PMG2), kneading (KNDG1 and KNDG2), and rotary evaporation (EVG1 and EVG2), respectively.

The X-ray diffraction (XRD) profiles of samples are shown in Figure 5. ABAME exhibited a single crystalline reflection at 14° (2θ) of great intensity, while *β*CD and *γ*CD exhibited several secondary crystalline reflections of medium to low intensity.

Systems produced with *β*CD in a 1:1 molar ratio (Figure 5A,C) showed a significant reduction in ABAME main crystalline reflection and were able to diffract X-rays in a similar way to CDs alone. However, in the inclusion complex (IC) prepared by rotary evaporation (EVB1), there was a greater suppression of crystalline reflections compared to the other systems. The same phenomenon occurred in systems produced using the 2:1 molar ratio (Figure 5B,D); however, due to the higher concentration of *β*CD, the suppression of ABAME crystalline reflections was more evident. With *γ*CD, a significant reduction in the main ABAME reflection was also observed, in addition to secondary reflections distinct from those of the individual components; this occurred mainly with the IC prepared with *γ*CD at the 2:1 molar ratio by rotary evaporation (EVG2), which showed medium-to-low intensity reflections at 7°, 14°, 15°, and 16° different from the other systems. These results suggest that the three proposed systems have a crystalline phase distinct from the isolated components, which is, therefore, an indication of complexation [19,20].

#### 2.2.2. Fourier Transform Infrared Spectroscopy

**Figure 6:**
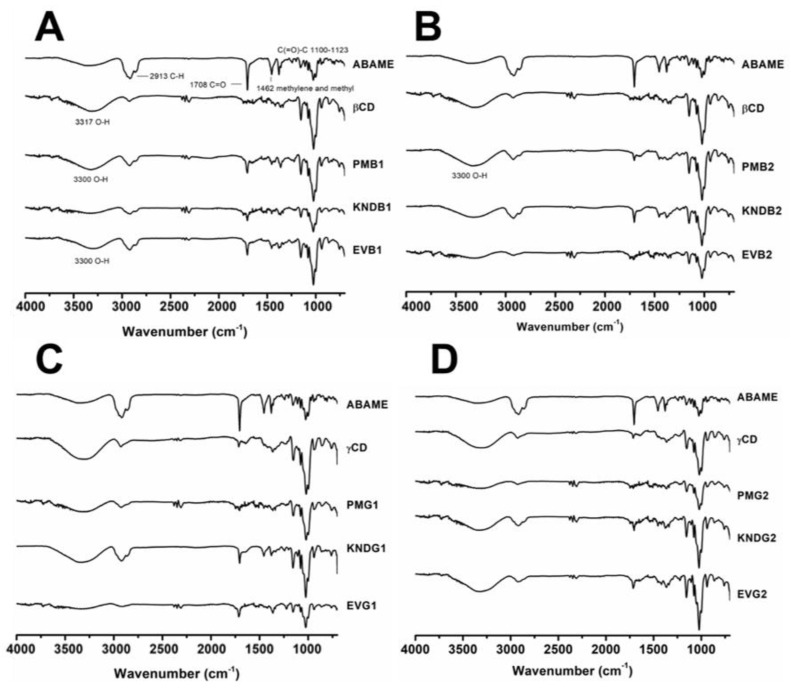
FTIR-ATR spectra of individual compounds, namely *α*,*β*-amyrenone (ABAME), *β*-cyclodextrin (*β*CD) and *γ*-cyclodextrin (*γ*CD), as well as inclusion complexes (ICs). ICs prepared with ABAME and *β*CD at 1:1 (**A**) and 2:1 (**B**) molar ratios using physical mixture (PMB1 and PMB2), kneading (KNDB1 and KNDB2), and rotary evaporation (EVB1 and EVB2), respectively. ICs prepared with ABAME and *γ*CD at 1:1 (**C**) and 2:1 (**D**) molar ratios using physical mixture (PMG1 and PMG2), kneading (KNDG1 and KNDG2), and rotary evaporation (EVG1 and EVG2), respectively.

Figure 6 shows the Fourier transform infrared spectroscopy (FTIR) spectra of the pure compounds and the different complexes obtained. It is possible to observe that the characteristic bands of cyclodextrins are predominant (axial deformation of the OH bond) around 3355 to 3300 cm^−1^. The spectrum obtained for ABAME is in agreement with the literature [1], with the appearance of an intense band of C=O bond at 1708 cm^−1^. The band observed at 2913 cm^−1^ can be assigned to vibrations of axial deformation of the C-H bond, which is characteristic of cyclic chains, while that at 1462 cm^−1^ can be assigned to the bending vibrations of the C-H bond of methyl or methylene groups. The spectra of the complexes showed similarities with the spectrum of *β*CD; that is, there was a masking of the ABAME bands in all samples. However, the bands around 3300 cm^−1^ in EVB1 and the IC prepared with *β*CD at the 2:1 molar ratio by physical mixture (PMB2) had greater intensity when compared to the other samples. A reasonable explanation is that the methods of rotary evaporation and physical mixture facilitated the inclusion of ABAME in the *β*CD cavity through a strong hydrogen bond between the guest and the host.

Similarly, the spectra of *γ*CD:ABAME complexes assumed the characteristics of CDs in both molar ratios. At 2250 and 3000 cm^−1^, the systems manifested the characteristics of the two compounds, pointing to a possible complexation.

#### 2.2.3. Scanning Electron Microscopy

**Figure 7:**
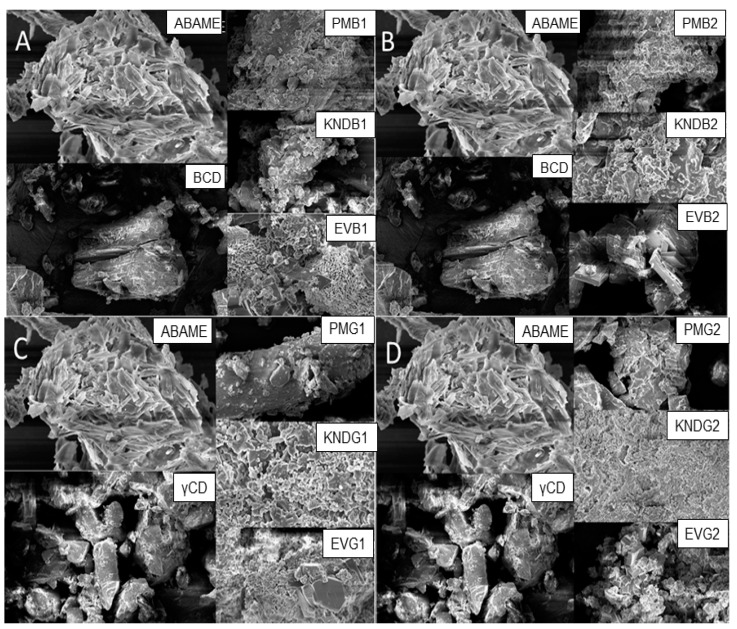
SEM micrographs of individual compounds, namely *α*,*β*-amyrenone (ABAME), *β*-cyclodextrin (*β*CD) and *γ*-cyclodextrin (*γ*CD), as well as inclusion complexes (ICs). ICs prepared with ABAME and *β*CD at 1:1 (**A**) and 2:1 (**B**) molar ratios using physical mixture (PMB1 and PMB2), kneading (KNDB1 and KNDB2), and rotary evaporation (EVB1 and EVB2), respectively. ICs prepared with ABAME and *γ*CD at 1:1 (**C**) and 2:1 (**D**) molar ratios using physical mixture (PMG1 and PMG2), kneading (KNDG1 and KNDG2), and rotary evaporation (EVG1 and EVG2), respectively.

The morphology of complexes obtained by physical mixture showed similarities with the individual ones of ABAME and cyclodextrin, being possible to observe the presence of crystals of both components in Scanning Electron Microscopy (SEM) images (Figure 7). On the other hand, it was not possible to observe the isolated compounds in samples obtained by the kneading method and rotary evaporation, demonstrating homogeneity, as seen by Gao et al. [21].

In *γ*CD, there were changes in the original structures, showing new forms; as can be seen in the complexes obtained by rotary evaporation, the prismatic structures when alone were replaced by cubic ones when complexed. In addition, even in the other systems, it was not possible to identify the individual structures of cyclodextrins and ABAME, indicating the effective formation of complexes.

As shown in Figure 7A, *β*CD appears as a prismatic crystal with edges, while the compound appears as an irregular crystal. SEM micrographs of the systems prepared by physical mixture were similar to the simple superposition of the compound and cyclodextrin, since both can be observed. On the other hand, Figure 7B shows a lesser degree of crystal masking in the IC prepared with *β*CD at the 2:1 molar ratio by rotary evaporation (EVB2), since crystals are evident in a less irregular shape and without porous appearance as in the previous one (Figure 7A), as well as the presence of blocks in the ICs prepared with *β*CD by kneading which were more compact at the higher (KNDB2) than at the lower (KNDB1) molar ratio.

The masking of the original structures also occurred in the ICs prepared with *γ*CD (Figure 7C), which showed changes in shapes. In particular, characteristics of the individual components cannot be identified in the IC prepared with *γ*CD by kneading at the 1:1 molar ratio (KNDG1), which suggests that the complex formed at the concentrations tested was the best. Moreover, Figure 7D referring to the complexes prepared by rotary evaporation shows that the structures at first prismatic were transformed into cubic ones. In this case, it is possible to identify the characteristics of the individual components only in samples prepared by physical mixing, indicating that there was the possible formation of the complex through evaporation and kneading techniques.

#### 2.2.4. Differential Scanning Calorimetry

**Figure 8:**
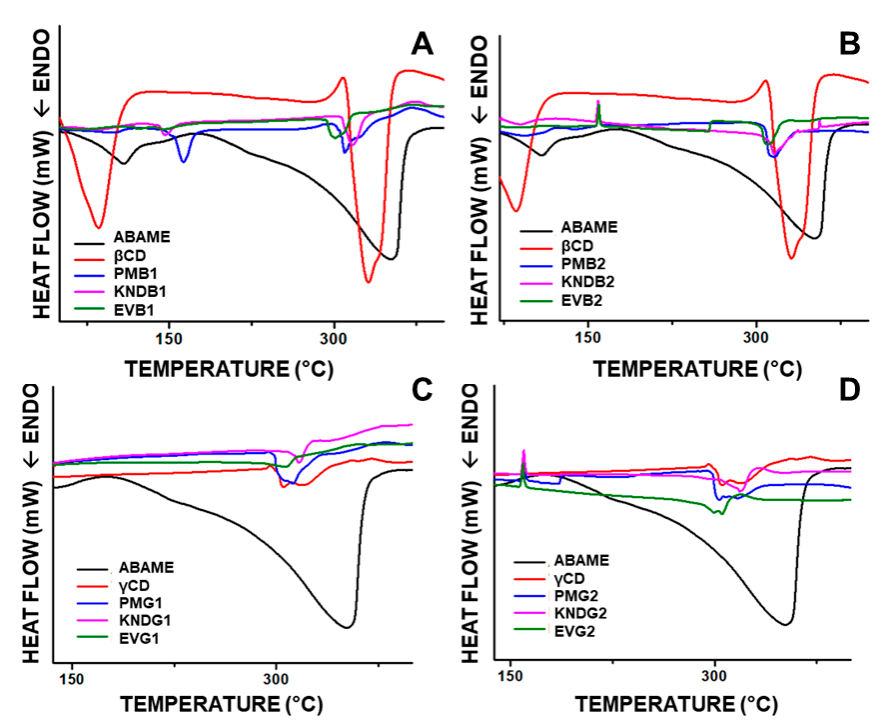
Differential Scanning Calorimetry curves of individual compounds, namely *α*,*β*-amyrenone (ABAME), *β*-cyclodextrin (*β*CD) and *γ*-cyclodextrin (*γ*CD), as well as inclusion complexes (ICs). ICs prepared with ABAME and *β*CD at 1:1 (**A**) and 2:1 (**B**) molar ratios using physical mixture (PMB1 and PMB2), kneading (KNDB1 and KNDB2), and rotary evaporation (EVB1 and EVB2), respectively. ICs prepared with ABAME and *γ*CD at 1:1 (**C**) and 2:1 (**D**) molar ratios using physical mixture (PMG1 and PMG2), kneading (KNDG1 and KNDG2), and rotary evaporation (EVG1 and EVG2), respectively.

The Differential Scanning Calorimetry (DSC) curve of ABAME (Figure 8) shows an endothermic event between 78 and 121 °C with a Tm of 108 °C, which was related to a variation of enthalpy (∆H) of 216.22 J g^−1^ and was possibly due to the melting of the two isomers. This event was followed by a second endothermic event, which coincided with the loss of mass through volatilization observed in the thermogravimetric analysis of ABAME alone [20].

In the sample prepared by physical mixture in the molar ratio of 2:1, there was an anticipation of the events mentioned above that occurred in the 1:1 ratio.

In addition, exothermic events occurred in complexes prepared at the 2:1 molar ratio (Figure 8B,D), which could be attributed to the characteristic recrystallization of ABAME in thermal analysis.

#### 2.2.5. Thermogravimetry

**Figure 9:**
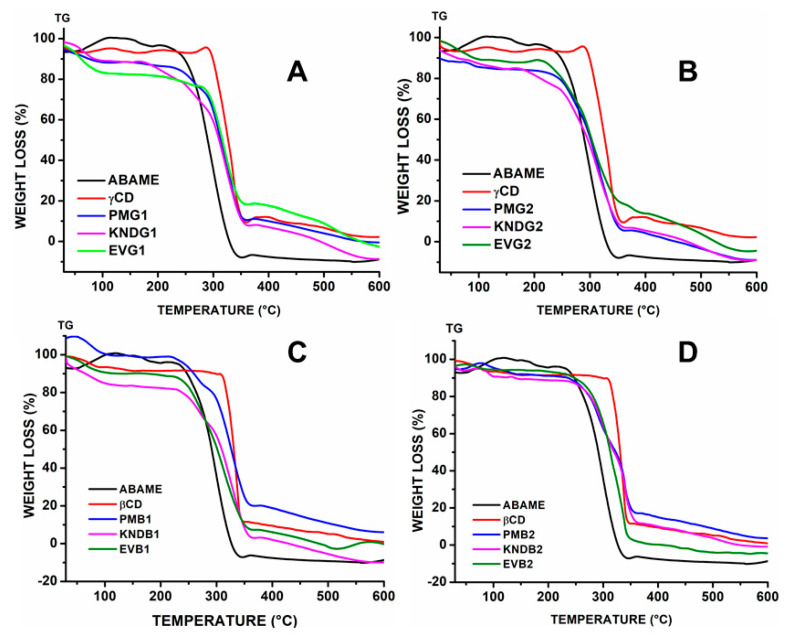
Thermogravimetry curves of individual compounds, namely *α*,*β*-amyrenone (ABAME), *β*-cyclodextrin (*β*CD) and *γ*-cyclodextrin (*γ*CD), as well as inclusion complexes (ICs). ICs prepared with ABAME and *β*CD at 1:1 (**A**) and 2:1 (**B**) molar ratios using physical mixture (PMB1 and PMB2), kneading (KNDB1 and KNDB2), and rotary evaporation (EVB1 and EVB2), respectively. ICs prepared with ABAME and *γ*CD at 1:1 (**C**) and 2:1 (**D**) molar ratios using physical mixture (PMG1 and PMG2), kneading (KNDG1 and KNDG2), and rotary evaporation (EVG1 and EVG2), respectively.

The thermogravimetry (TGA) curve of ABAME shows only a well-defined stage of mass loss related to its volatilization (Figure 9). The percentage of mass loss (Δm%) was about 99.5% and 99.5% with initial and final temperatures of 330 and 520 °C, respectively. The results showed triterpene high thermal stability, with mass loss in a single step, starting at 330 °C and ending at 425 °C, with approximately 93% of Δm%. The CI’s TGA curves evidenced no significant variation in the ABAME thermal stability, which remained stable in the same degradation temperature range. Only EVG1 was shown to require a higher temperature for the beginning of mass loss. Additionally, a major decomposition process was observed between 400 and 520 °C followed by carbonization.

### 2.3. In Vitro Activity

#### Inhibition of Lipase Activity

**Figure 10:**
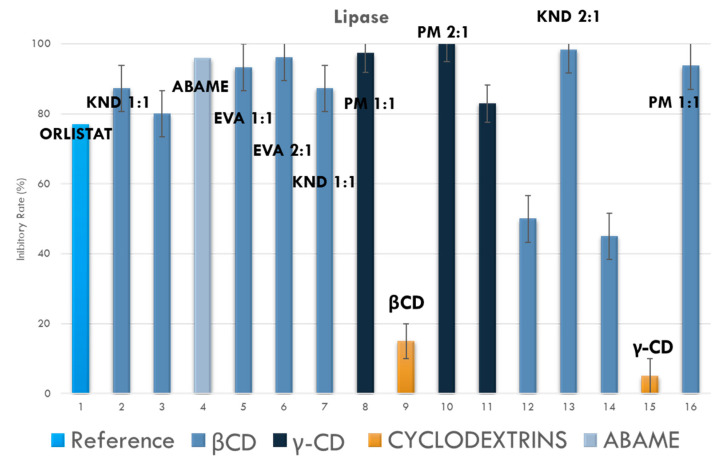
Rate of inhibition of lipase activity (%) exerted by different samples at a concentration of 1 µg mL^−1^. Data, worked out by the statistical software GraphPad Prism (version 6.0), are expressed as means ± SD. *p* < 0.05 was considered statistically significant when compared to the control group.

Figure 10 shows the rate of inhibition of lipase activity (%) exerted by the different samples, labelled from 1 to 15, at a concentration of 1 µg mL^−1^, while Table 4 lists the corresponding IC_50_ (± SD) values for lipase inhibition rates higher than 50%.

In Table 4, only the IC_50_ values of samples (1, 3, 4, 6, 7, 9 and 12) that significantly inhibited lipase in cells of porcine type II Sigma Aldrich have been listed. On the other hand, samples 2, 10, 11, and 13 refer to complexes that did not provide relevant values for this analysis. Upon comparing the activity of ABAME alone with IC_50_ 95.9 ± 1.9 and the activity of the inclusion complexes, the samples showed inhibition factors ranging from 87.3 ± 1.7 to 100.3 ± 3.4.

## 3. Discussion

### 3.1. Computational Theoretical Study

#### 3.1.1. Physicochemical Properties In Silico (SwissADME)

Table 1 shows that all values in the docking obtained were negative, with an average of −8.20 among the three parameters, namely ESOL-7,99, ALI-9,0, and SILICOS-IT-7,63, which indicates a poorly water-soluble compound.

The average permeability, determined by Darcy’s Law, was −2.66 cm s^−1^. Since the closer the values are to 1, the more permeable the compound is on the skin, the data obtained suggest the need for complexation to improve this characteristic [22].

In addition, Lipinski et al. [23], in their pioneering work on active compounds, defined ranges of values of physicochemical parameters for a high probability of being oral drugs (that is, similarity to drugs) and defined the so-called “rule of five”, which is capable of delineating the relationship between pharmacokinetics and physicochemical parameters and indicating whether an insoluble molecule could be a drug to be administered orally. The binary mixture of *α*,*β*-amyrenone showed few violations demonstrating similarities with drugs already marketed and administered orally.

#### 3.1.2. Virtual Screening

In Virtual Screening, a database with target structures is built for coupling the tested molecule based on the characterization and grouping of the shape of the binding site, in order to increase the rate of success of selective inhibitors for the desired target protein through the process [24]. In the process, five receptors were identified with activities that could correlate with the possible action of ABAME. The best receptor was the PPAR-*γ* one, which is a receptor for pancreas enzymes and already consolidated drugs that aim at anti-obesity treatment and tend to have drug-binding action on it. The second highest affinity was identified for the 6Q5 ligand. In this case, the type of target receptor was changed, it being a ROR receptor, that is, an orphan receptor related to RAR. The members of this family are nuclear receptors for intracellular transcription factors, whose natural identity is still controversial, but it is already reported that they are linked to oxysterols, some of which in position 7 may be agonists with high lipid affinity [25,26,27,28]. According to Solt and Burris [26], RORs can function as lipid sensors and, therefore, they can play a role in regulating lipid metabolism, which corroborates the highest affinity predicted in this study. The other receptors with less affinity are also related to the metabolic system. Among them, the OSC receptor is related to oscillarin inhibition, playing a role in the coagulation of thrombosis. A8W, similar to pregnenolone sulfate, a precursor to steroid hormones targeting GABA_A_, is related to neurotransmitters. Finally, the 4RX targeting BACE-1 is used as a target for diseases such as Alzheimer’s.

#### 3.1.3. Molecular Docking

In recent years, one of the most important advances in the development of medicines has been the implementation of in silico methodologies [29]. The Molecular Docking methodology explores the behavior of small molecules at the binding site of a target protein. As more protein structures are determined experimentally using X-ray crystallography or nuclear magnetic resonance, Molecular Docking is increasingly used as a tool in the discovery of new drugs [30,31].

For host–guest interaction between CDs and the drug, no covalent bonding occurs. However, due to the action of weak non-covalent forces, such as hydrogen bonds, hydrophobic interactions, and van der Waals forces, the host molecules are forced to remain inside the CD cavity, keeping the entire system in equilibrium [32,33]. Through the host–guest interaction with CDs, remarkable modifications of different physicochemical and/or biological characteristics of the guest molecules can be achieved [14,32].

The energies were minimized in each step, and for each angle, and the results of the theoretical computational study are shown in Figure 1 and Figure 2. For docking with *β*-cyclodextrin and *γ*-cyclodextrin, energies of −40.24 and −21.07 kcal mol^−1^ were calculated, respectively. The molecular modeling study also showed that the best complex would be the one prepared using *β*-cyclodextrin in a 2:1 molar ratio, which showed the most negative energy among those tested, indicating the greatest stability at this ratio of concentrations.

### 3.2. Physicochemical Characterization

#### 3.2.1. X-ray Diffraction

The powder X-ray diffraction technique makes it possible to verify whether the new chemical entity obtained shows differences in the solid phases in relation to the original solid drug, although in most cases, the solid inclusion complexes are non-crystalline [34]. In this respect, the significant reduction of the main crystalline reflection of ABAME with both proposed CDs, combined with the appearance of new crystalline reflections different from the original components, are indicative of the formation of a new three-dimensional pattern of the arrangement of system atoms, that is, of a new crystalline phase and, therefore, of complexation [19,20].

#### 3.2.2. Fourier Transform Infrared Spectroscopy

The Fourier transform infrared spectroscopy technique is widely used in the characterization of solid systems with CDs, as it is fast and accurate, but the information it provides is limited [35]. Nonetheless, this technique is very useful to identify changes in the characteristic bands of the vibrational pattern of the ligand and CD, such as disappearance, enlargement, change in peak intensity, or deviations in its wavenumber, which can be a strong indication of the interaction between ligand and CD [36].

The formation of inclusion complexes (ICs) in the solid phase can be evaluated by comparing the infrared spectra of the pure drug and the solid complexes obtained by different preparation methods [37,38,39]. When an IC is formed, small displacements of CD bands can mask the characteristic ones of the ligand, because its encapsulation occurs in the CD internal cavity via hydrophobic interactions and Van der Waals forces. However, if the ligand has characteristic functional groups such as a carbonyl, the band may be significantly masked and sometimes displaced after complexation [12,20,39,40].

In all spectra, the ABAME bands were masked, and the intensity of the band at 3500 cm^−1^, which is characteristic of CDs [41], decreased in those of complexes, which indicates ABAME inclusion in the CD cavity suggested by the computational simulation.

#### 3.2.3. Scanning Electron Microscopy

Scanning electron microscopy (SEM) has traditionally been used in the biomedical sciences to characterize the topography of the cell and tissue surfaces [42]. In addition, although several techniques are available to measure the size and shape of particles, those based on electron microscopy are often considered to be the preferred methods for characterizing their dimensional properties [43]. The results above showed that the morphology of the ABAME-incorporating preparations is different from that of CDs, indicating the possible formation of ICs [21].

As already shown in Figure 7, *β*CD appeared as a prismatic crystal with edges, while ABAME appeared as an irregular crystal. SEM micrographs of samples prepared by physical mixture showed a simple overlap of ABAME and cyclodextrin, with the presence of both compounds being evident, while the morphology and particle size of ICs were completely different from each other. The morphology of the *β*CD/ABAME IC was similar to that of *β*CD, corroborating the XRD findings. In addition, the IC showed several irregularly shaped crystals with loose surface bonds, and the original state of CD and ABAME could not be observed to a considerable extent [21]. Using *γ*CD, in addition to masking the original structures, there were changes in shapes, as can be seen in the systems obtained by evaporation, in which the structures, initially prismatic, were transformed into cubes. Finally, not even using the other preparation techniques, it was possible to detect in the ICs the structures of CD and ABAME individually.

#### 3.2.4. Differential Scanning Calorimetry

The occurrence of exothermic events in ICs prepared using the 2:1 molar ratio suggests recrystallization. When the samples are only partially crystalline, the Bragg peaks are smaller and overlapped. It must be taken into account that the tendency to crystallization clearly depends on the formulated pharmaceutical ingredient, more specifically on its ability to form crystals [44,45]. Changes in the thermal stability of a drug can also be an indication of inclusion, being evidence of the formation of a new supramolecular structure characteristic of ICs [46,47].

In previous studies, the class of terpenes also manifested recrystallization events when coupled with cyclodextrins. It has been stressed that this is a characteristic of the compound when subjected to couplings with improving agents [48,49]. According to other authors, the phenomenon of recrystallization occurs simultaneously during deformation [50,51], showing similarities with previous studies where the compounds managed to enter totally or partially in the cyclodextrin cavity and remained with some stability.

When an inclusion complex is formed and analyzed by thermal analysis, masking of the drug or bioactive molecule melting point is often observed [52]. However, in cases such as this, in which the ABAME melting point was still preserved, there may have been a partial coupling of the molecule with CD; that is, part of the molecule may have remained outside the system. This event may be the result of manual preparation of the complex and the guest–host size relationship, so that the virtual model is not always able to fully follow reality, and the connections occur in different ways, not excluding the efficiency of the complex.

#### 3.2.5. Thermogravimetry

Thermogravimetric (TGA) analysis is a technique in which the mass of the sample is continuously measured as a function of temperature, that is, as it is heated or cooled [53].

For ABAME, no major differences were observed between the TGA curves of samples prepared by physical mixture (PM), kneading (KND), and rotary evaporation (RE), although the first ones showed less thermal stability compared to the others. The TGA curves of samples prepared by RE (Figure 9) showed a loss of initial mass due to water. In addition, samples prepared by KND and RE showed better results than those prepared by PM, with the exception of the *γ*CD complex ones prepared in a 2:1 molar ratio that maintained their thermal stability. However, samples prepared by PM with *β*CD ensured greater ABAME thermal stability.

### 3.3. In Vitro Activity

#### Inhibition of Lipase Activity

Lipases secreted by the pancreas and salivary glands are key enzymes for the absorption of triglycerides in the small intestine, and the ability to inhibit them is one of the most widely used criteria to determine the potential of a natural product as an anti-obesity agent [54]. Orlistat is a potent inhibitor of pancreatic and gastric lipases, which are the enzymes responsible for the hydrolysis of lipids in free fatty acids and monoacylglycerols, the use of which can reduce the absorption of dietary fat by approximately 30% [55].

Several phytochemical constituents of medicinal plants have been studied for their ability to inhibit lipases and thus treat obesity, among which are saponins, polyphenols, and terpenes [56]. Terpenes are a family of chemically diverse compounds found in nature. Many terpenes produced by plants have important pharmacological activities, including anti-tumor, choleretic, sedative, analgesic, anti-inflammatory, anti-helminthic, cardioprotective, antioxidant, anti-allergic, gastroprotective, hypoglycemic, antihypertensive, and antimicrobial activities [57,58,59,60].

Among the studies with triterpenes as anti-obesity agents, we called attention to the pentacyclic triterpenes [61], the class to which the ABAME mixture belongs. In a previous study, ABAME alone allowed a percentage of lipase inhibition as high as 80% [1], and its complexes with cyclodextrins potentiated such an inhibitory effect being able to ensure almost equivalent performance (about 70% of activity on average) with lower concentration of the active ingredient.

These results showed that this molecule has great potential for the development of new pharmaceutical forms and new drug delivery systems. Thus, this compound can be an effective choice for the development of formulations that can be used to treat type II diabetes, metabolic syndrome, and obesity disorders. This is mainly because it presents an improvement in its results when compared to the isolated compound, indicating the improvement in stability and solubility, with a better systemic activity.

## 4. Materials and Methods

### 4.1. Materials

The binary mixture of *α*,*β*-amyrenone (ABAME) was obtained by the oxidation of commercial oleoresin from *Protium heptaphyllum* isolated in the state of Amazonia, AM, Brazil. The reaction yield based on 1.0 g of *α*,*β*-amyrin was about 70% of *α*,*β*-amyrenone with approximately 0.9966 purity [1]. Cyclodextrins (CDs) were purchased from Sigma Aldrich (St. Louis, MO, USA), while analytical grade solvents were used for analyses.

### 4.2. Theoretical Computational Studies

#### 4.2.1. Physicochemical Properties In Silico (SwissADME)

Chemical structures of *α*-amyrenone and *β*-amyrenone were previously designed on the SwissADME platform using Marvin JS (ChemAxon, Budapest, Hungary). Then, the physicochemical parameters, lipophilicity, solubility, and oral viability were calculated.

#### 4.2.2. In Silico Screening to Elucidate the Site of Action (Virtual Screening)

The structure of *α*- and *β*-amyrenone was drawn using the program MarvinSketch 16.9.5 (ChemAxon, Budapest, Hungary). The program Avogadro was used to obtain the 3D model of the compound and the required files for inverse Virtual Screening method [62]. After this initial process, the geometry was optimized using the semi-empirical method PM7 implemented in the MOPAC2016 software [63].

The ligand-based inverse Virtual Screening was carried out to find molecular receptors potentially responsible for the mechanism of action described for *α*-amyrenone. For this purpose, a ligand library was constructed from the RCSB PDB Protein Data Bank [64], which comprised more than 26,000 compounds. The method was performed by 3D molecular similarity calculation between *α*-amyrenone and the compounds of the library. This process was made using the ShaEP algorithm [65] and automated ad hoc by shell scripting. To rank the results, we considered only the highest similarity scores.

#### 4.2.3. Molecular Docking

The 3D models of the *α*-amyrenone and *β*-amyrenone ligands were designed separately using the MarvinSketch 18.16 software (ChemAxon, Budapest, Hungary). These molecules were subjected to coupling simulations with *β*-cyclodextrin and *γ*-cyclodextrin. In addition, all complexes were optimized through the PM6-DH + semi-empirical theory level using the MOPAC program. The results of the conformations of these complexes (cyclodextrin–ligand) were analyzed visually by the UCSF-Chimera software. A binary mixture in solid form of the two compounds was used in the experimental tests, while in in silico studies the three-dimensional structures of the two compounds complexed with cyclodextrins were analyzed separately, their respective energies were calculated, and an average value of them was presented.

### 4.3. Preparation of Inclusion Complexes

The inclusion complexes (ICs) were prepared by the methods of physical mixing (PM), kneading (KND), and rotary evaporation (RE), using in all cases molar ratios of 1:1 and 2:1 CD:ABAME and considering molecular weights of ABAME, *β*-cyclodextrin (*β*CD), and *γ*-cyclodextrin (*γ*CD) of 849.40, 1134.98, and 1297.12 g mol^−1^, respectively.

#### 4.3.1. Physical Mixture

The individual components (ABAME, *β*CD, and *γ*CD) were weighed separately based on the molar ratio. ABAME and CDs were mechanically homogenized in porcelain grit with the aid of a pistil, and the resulting powder was kept in a desiccator until further analysis. Samples prepared by PM in the 1:1 molar ratio were named PMB1 and PMG1 and those in the 2:1 molar ratio were named PMB2 and PMG2 when *β*CD or *γ*CD was used, respectively.

#### 4.3.2. Kneading

The KND method was applied according to Galvão et al. [66] with modifications. ABAME and CDs were weighed separately, added to a porcelain mortar, and homogenized with the aid of a pistil, as described in Section 4.3.1. Then, a mixture of distilled water/ethanol (20:80) was added to the resulting powder until a paste was formed. Finally, this paste was dried in an oven at 60 °C. The product was collected and stored in a desiccator for future analyses. Samples prepared by KND in the 1:1 molar ratio were named KNDB1 and KNDG1, and those in the 2:1 molar ratio were named KNDB2 and KNDG2 when *β*CD or *γ*CD was used, respectively.

#### 4.3.3. Rotary Evaporation

ABAME and CDs were weighed separately according to the desired molar ratio and sprayed in grains with the aid of a pistil. Then, a volume of ethanol/distilled water was gradually added until complete solubilization. The resulting solution was subjected to drying for 30 min in a rotary evaporator, model RV10 (IKA, Fullerton, CA, USA), operating at 150 rpm. After that, the sample obtained was dried at 50 °C in an oven for 24 h. Samples prepared by RE with the 1:1 molar ratio were named EVB1 and EVG1, and those with the 2:1 molar ratio were named EVB2 and EVG2 when *β*CD or *γ*CD was used, respectively.

### 4.4. Physicochemical Characterization of Inclusion Complexes

To detect the formation of ICs between ABAME and CDs, samples were physicochemically characterized by Fourier transform infrared spectroscopy (FTIR), scanning electron microscopy (SEM), powder X-ray diffraction (XRD), thermogravimetric analysis (TGA), and differential scanning calorimetry (DSC) [20,67].

#### 4.4.1. Powder X-ray Diffraction

The XRC profiles of samples were obtained on a diffractometer, model D2 Phaser (Bruker, Billerica, MA, USA), using CuK*α* radiation (λ = 1.54 Å) with a Ni filter. The analysis was performed with 0.02° step size, 10 mA current, 30 kV voltage, and using the Lynxeye detector.

#### 4.4.2. Fourier Transform Infrared Spectroscopy

The FTIR analysis was carried out in the wavenumber region of 700 to 4000 cm^−1^, with 20 scans and resolution of 4 cm^−1^ using an attenuated total reflectance accessory (ATR/FTIR) in a spectroscope, model IR Prestige-21 (Shimadzu, Tokyo, Japan).

#### 4.4.3. Scanning Electron Microscopy

Samples were heated to 40 °C for 24 h, metallized in gold for better visualization of the structure, and distributed in the support (stub) of the microscope with adhesive tapes. The analyses were performed in an electron microscope, model Tabletop Microscope TM3000 (Hitachi, Tokyo, Japan), with a magnitude of 2000×. The photomicrographs were obtained with an acceleration potential of 15 kV under reduced pressure.

#### 4.4.4. Differential Scanning Calorimetry

DSC analyses were performed on a calorimeter, model DSC-50 (Shimadzu), using approximately 2 mg of sample positioned in aluminum crucibles, under a dynamic nitrogen atmosphere (50 mL min^−1^), heating rate of 10 °C min^−1^, and temperature range of 30–600 °C. The equipment was calibrated with indium (melting point: 156.6 °C; ΔHfus. = 28.54 J g^−1^) and zinc (melting point: 419.6 °C).

#### 4.4.5. Thermogravimetry

The TGA profiles were obtained in a thermobalance, model TGA-60 (Shimadzu). Samples were subjected to heating in the temperature range between 30 and 600 °C. Approximately 2 mg of sample were added in alumina crucibles, which were heated under a dynamic nitrogen atmosphere (50 mL min^−1^) and heating rate of 10 °C min^−1^. The thermobalance was calibrated using a CaC_2_O_4_.H_2_O standard in accordance with ASTM.

### 4.5. In Vitro Experiments on Lipase Activity

Samples were initially dissolved in 10 µg mL^−1^ dimethyl sulfoxide (DMSO) and then diluted to 1 µg mL^−1^. The final concentration of DMSO did not exceed 0.2%.

Lipase activity was determined according to Slanc et al. [68] with modifications. Pancreas porcine lipase Type II (Sigma-Aldrich Brasil, São Paulo, SP, Brazil) was diluted in TRIZMA^®^ Base buffer (Sigma-Aldrich Brasil) at 75 mM, pH 8.5. *p*-Nitrophenyl palmitate (PNP) used as a substrate (Sigma-Aldrich Brasil) was diluted in acetonitrile and then in ethanol in the proportion of 1:4.

Orlistat (Sigma-Aldrich Brasil) was used as a standard. Readings were performed on a microplate reader, model DTX 800 Multimode Detector (Beckman Coulter, Lane Cove, NSW, Australia), at 450 nm. Then, 30 µL of test samples of the standard and/or control (DMSO) were placed in different wells of the microplate in triplicate. Then, 250 µL of lipase solution (0.8 µg/mL) were added. The mixture was kept incubated for 5 min at 37 °C under dark conditions; then, 20 µL of PNP (4 µg mL^−1^) were added. Readings were taken every 10 min or until the control reading reached an absorbance of 1000 ± 0.1.

After determining the percentage of inhibition, samples with inhibition greater than 50% were diluted in serial concentrations (100, 80, 60, 40, 20, 10, and 5 µg mL^−1^) to determine the IC_50_ (%).

#### Statistics

The results of the lipase inhibition rate determined in triplicate for samples of the *β*CD, *γ*CD, isolates, and ABAME groups as well as the control (Orlistat) were submitted to statistical analysis by the *t*-test. Results were expressed as mean ± SEM, with statistical significance set at * *p* < 0.05 compared to the control group. The GraphPad Prism Software (version 6.0) was used to perform the analyses.

## 5. Conclusions

Inclusion complexes (ICs) of *α*,*β*-amyrenone (ABAME) with *γ*- and *β*-cyclodextrins (CDs) were successfully prepared in the present work. The in silico prediction by molecular modeling corroborated the results obtained by physicochemical characterization and in vitro tests. In addition, the in silico study was able to show the theoretical physicochemical profile of the molecule, confirming its low solubility in water. In line with this study, Virtual Screening was able to elucidate the possible receptors for coupling the compound in the metabolism, indicating that it is a binary mixture with affinity to the lipid metabolism receptors. After Molecular Docking study, it was possible to develop ICs with *β*CD and *γ*CD by different methods, with the most stable systems in silico being those prepared using a 2:1 CD:ABAME molar ratio. The FTIR analysis indicated masking of the characteristic bands of *α*,*β*-amyrenone, while the results of XRD and SEM showed the formation of a more stable system than the individual components, with a new crystalline phase and a morphological aspect distinct from the original compounds. In vitro tests of lipase activity inhibition showed that both ABAME alone and complexes have a desirable performance against pancreatic lipases. However, the anti-obesity activity of complexes was higher than that of the triterpene alone, which would require higher concentrations to be successful. In conclusion, complementary in vivo tests are still necessary so that the proposed formulations can be considered a potential alternative drug for the treatment of obesity.

## Figures and Tables

**Table 1:** Physicochemical properties, solubility, and lipophilicity profiles, and similarity to medicines (druglikeness) according to SwissADME.

Molecular Formula		Molecular Weight		Molar Refractivity	
C_30_H_48_O		424.70 g/mol		134.05	
**Lipophilicity (log P)**					
ILOGP	XLOGP3	WLOGP	MLOGP	SILICOS-IT	Consensus log P
4.52	8.76	8.30	6.82	7.31	7.14
**Solubility in water (log S)**					
ESOL		ALI	SILICOS-IT	RESULT	
−7.99		−9.0	−7.63	Low solubility	
**PHARMACOKINETICS**					
log Kp (skin permeation)				−2.66 cm s^−1^	
DRUGLIKENESS					
Lipinski			1 Violation: MLOGP > 4.15		
Ghose			2 Violations: WLOGP > 5.6 and MR > 130		
Egan			1 Violation: WLOGP > 5.88		
Muegge			2 Violations: XLOGP3 > 5 and Heteroatoms < 2		
Bioavailability			0.55		

**Table 2:** Ligands with greater structural similarity to the binary mixture of *α*,*β*-amyrenone.

Ligand PDB ID	Similarity	Target
5VN	0.8912	PPAR-*γ*
6Q5	0.8797	ROR
LAN	0.8156	OSC
A8W	0.7875	GABAA
4RX	0.7836	BACE-1

**Table 3:** Computed average values of intermolecular interaction energy.

Energy (kcal.mol^−1^)	Molar Ratio	Type of Cyclodextrin
−40.24	1:1	*β*-
−21.07	1:1	*γ*-
−109.67	2:1	*β*-
−81.87	2:1	*γ*-

**Table 4:** IC50 ± SD values of samples in serial concentrations that showed lipase (porcine type II Sigma Aldrich) inhibition higher than 50%.

Sample	IC_50_ ± SD (µg mL^−1^)	Sample	IC_50_ ± SD (µg mL^−1^)
Orlistat	0.77 ± 0.02	6	87.3 ± 0.72
1	87.3 ± 1.7	7	97.3 ± 4.3
3	95.9 ± 1.9	9	100.3 ± 3.4
4	93.3 ± 4.2	12	98.3 ± 3.1

## Data Availability

The data presented in this study are available on request from the corresponding author.

## Patents

BR 10 2020 014505 3. Deposited on 16 July 2020.
